# Mechanics of fragmentation of crocodile skin and other thin films

**DOI:** 10.1038/srep04966

**Published:** 2014-05-27

**Authors:** Zhao Qin, Nicola M. Pugno, Markus J. Buehler

**Affiliations:** 1 Laboratory for Atomistic and Molecular Mechanics (LAMM), Department of Civil and Environmental Engineering, Massachusetts Institute of Technology, 77 Massachusetts Ave., Room 1-239, Cambridge 02139, MA, USA; 2 Center for Computational Engineering, Massachusetts Institute of Technology, 77 Massachusetts Ave., Cambridge, MA 02139, USA; 3 Laboratory of Bio-Inspired & Graphene Nanomechanics, Department of Civil, Environmental and Mechanical Engineering, Università di Trento, via Mesiano, 77 I-38123 Trento, Italy; 4 Center for Materials and Microsystems, Fondazione Bruno Kessler, Via Sommarive 18, 38123 Povo (Trento); 5 School of Engineering & Materials Science, Queen Mary University of London, Mile End Road, London E1 4NS, UK; 6 Center for Materials Science and Engineering, Massachusetts Institute of Technology, 77 Massachusetts Ave., Cambridge, MA 02139, USA

## Abstract

Fragmentation of thin layers of materials is mediated by a network of cracks on its surface. It is commonly seen in dehydrated paintings or asphalt pavements and even in graphene or other two-dimensional materials, but is also observed in the characteristic polygonal pattern on a crocodile's head. Here, we build a simple mechanical model of a thin film and investigate the generation and development of fragmentation patterns as the material is exposed to various modes of deformation. We find that the characteristic size of fragmentation, defined by the mean diameter of polygons, is strictly governed by mechanical properties of the film material. Our result demonstrates that skin fragmentation on the head of crocodiles is dominated by that it features a small ratio between the fracture energy and Young's modulus, and the patterns agree well with experimental observations. Understanding this mechanics-driven process could be applied to improve the lifetime and reliability of thin film coatings by mimicking crocodile skin.

Skin fragmentation (also known as crocodile cracking), is a common way for thin films to release deformation energy that is caused by tension^1^,^2^,^3^. This process is irreversible and leaves complex pattern as many cracks interact and go through the material surface. The geometry and size of those fragmentation patterns varies dramatically for different materials including organic and inorganic systems such as fruit skin, paintings and ceramics^4^,^5^, as well as a nanofilm with atomic thickness^6^ (Fig. 1). They are generally considered a threat to engineering systems. For instance, steel and wood structures in buildings and turbines with fragmented coating materials are largely exposed to environment and subjected to corrosion. It is critical to understand the mechanism behind such phenomena crossing multiple scales. Especially, how tensile forces initiate the fragmentation in thin films and what material characteristic stabilizes the pattern remain largely illusive. Fundamental answering of those questions can help the design of surface coating with improved lifetime and resistance.

The fragmented skin on a crocodile's head provides a suitable case to investigate the skin fragmentation. The skin acts like scales of snakes, lizards and fishes^7^,^8^ to protect the crocodile, but instead of gene regulation, its pattern is induced by skeletal growth during the embryonic stage^9^. This contradicts with the conventional thinking, suggesting that some fragmentation may actually enhance the protection function. Moreover, it is intriguing, but unclear, what is the mechanical basis for crocodiles to generate this skin fragmentation with a characteristic size. By learning from the fragmentation of crocodile skin, we expect to gain knowledge in improving the design of engineered surface coatings.

In our current study, we combine theoretical analysis with numerical simulation that uses a simple thin film model for the crocodile skin. We note that prior studies have investigated the fragmentation in surface coatings using numerical methods, but most of them require pre-existing defects for crack initiation^10^,^11^,^12^,^13^. It is difficult to decouple the effect of random distributions of weak regions and the effect of material property on the characteristic of fragmentation pattern by using those models. Here, we use a simple elastic network model with a uniform spring stiffness and strength (detail in the **Methods** section), without additional defects, to investigate how the characteristic size of fragmentation is affected by mechanical properties of the film material and loading conditions. It is shown that by adjusting the spring characteristic we can reproduce the characteristic of fragmentation pattern as is found in different materials including crocodile skin.

## Results

**Figure 1:**
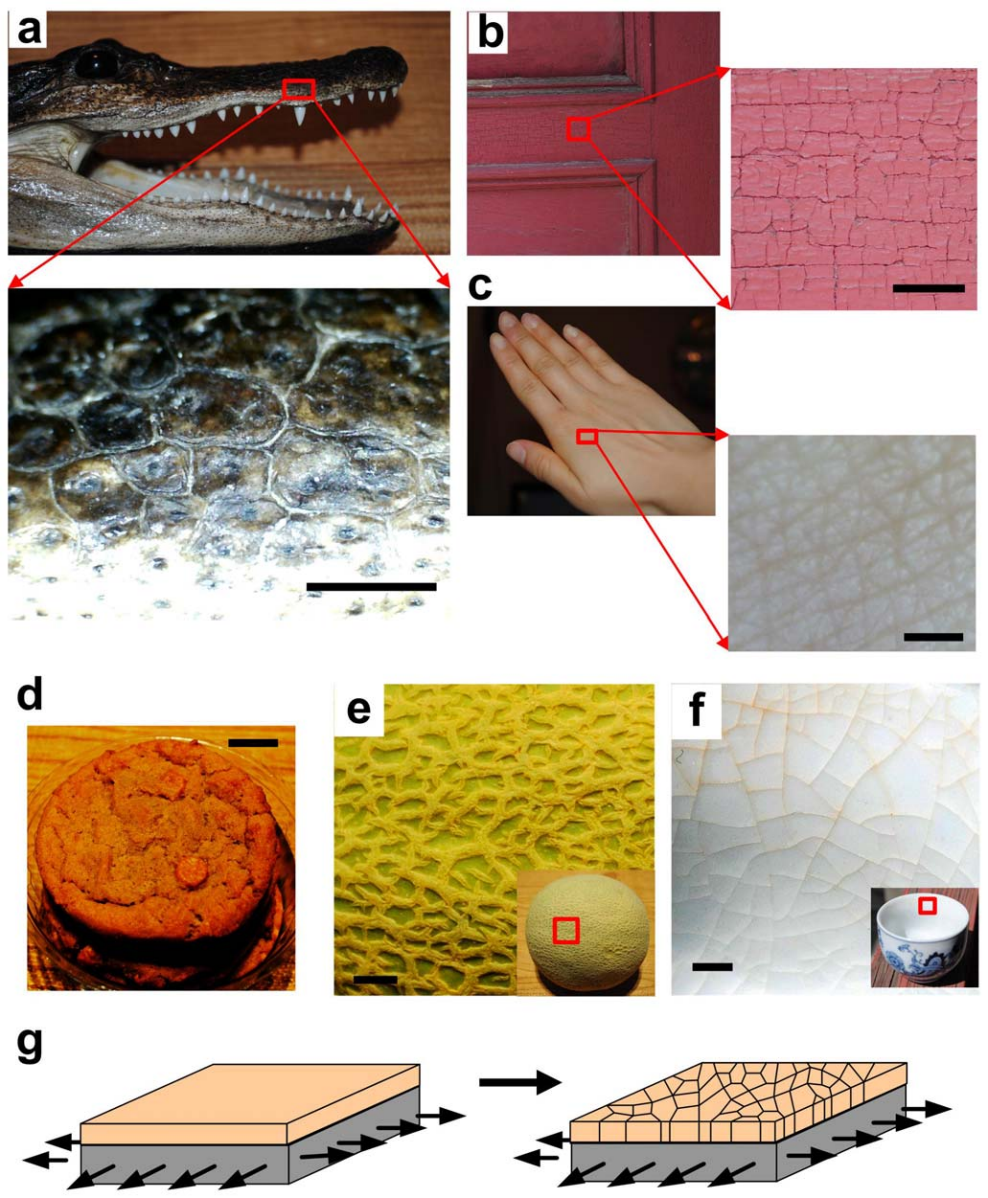
Widely observed skin fragmentation patterns in animals, paint, skin, food, plants and ceramics. (a), Snapshot of a crocodile head. Bottom: close-up of skin fragmentation in the form of scales on a crocodile head surface (the crocodile is small with an eye to nose distance of 90 mm). Scale bar: 4 mm [Boston, MA, image credit to ZQ]. (b), Skin fragmentation observed on the surface of painted door [Boston, MA, image credit to ZQ]. Scale bar: 1 cm. (c), Skin crumpling observed on the back of a hand. Scale bar: 2 mm [Boston, MA, image credit to ZQ]. (d), Skin fragmentation observed on cookie surface. Scale bar: 16 mm [Boston, MA, image credit to ZQ]. (e), Skin fragmentation pattern observed on a cantaloupe surface. Scale bar: 6 mm [Boston, MA, image credit to ZQ]. (f), Skin fragmentation observed on the inner surface of a porcelain cup [Boston, MA, image credit to ZQ]. Scale bar: 3 mm. (g), Schematic of the mechanical model used to model skin fragmentation under deformation. The overarching question for those phenomena is how tensile forces initiate the fragmentation and what material characteristic determines the geometry of the patterns as we observe on different surfaces.

**Figure 2:**
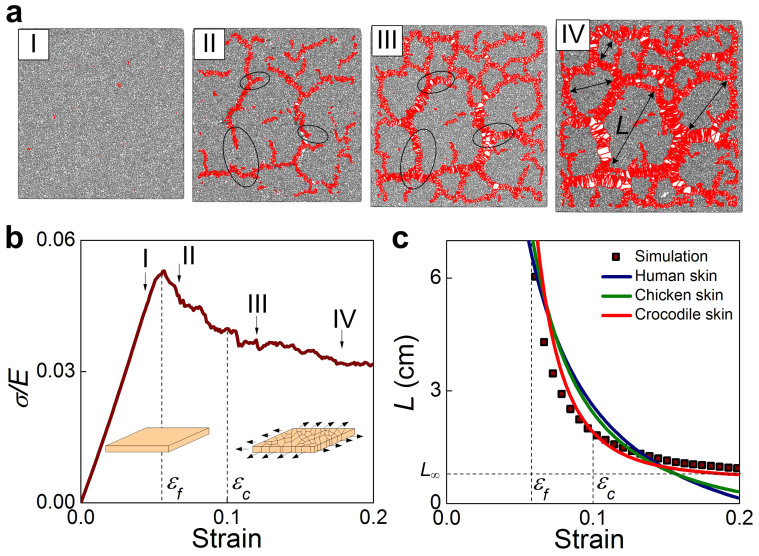
Illustration of the process of fragmentation under biaxial tensile deformation. (a), The increasing strain leads to evolving cracking patterns in different stages; (I) no crack, (II) crack growth, (III) forming a stationary network of cracks, and (IV) further opening of existing cracks without newly formed cracks. (b), The overall strain-stress relation during deformation, with the four stages shown in (a) marked on the graph. (c), The increasing strain leads to growing fragments with decreased size, yielding an asymptotic size of *L*_∞_ when the material is fully fractured (*i.e.* stage IV depicted in a) and no further changes in fragmentation pattern are induced by strain increments. The continuum curves are fitted according to Eq. (3) by using 2*γ*/*E* = 3 × 10^−1^, 4 × 10^−2^ and 2 × 10^−4^ cm for human hand skin, chicken skin and crocodile skin (finding α = 2.4), respectively. The typical Poisson's ratio, *ν* = 1/3, is used here. The agreement between the simulation result and the fitted curve based on the mechanics of crocodile skin suggests that the asymptotic size of fragmentation is dominated by the small ratio between the fracture toughness and stiffness.

Here we use a simple network model to study the fragmentation behavior of the thin film as depicted schematically in Fig. 1g (details see **Methods section**). We apply biaxial tensile strain to this model to investigate how fragmentation generates and grows. The fragmentation pattern is identified as an assembly of polygons as shown in Fig. 2a. Notably, cracking only appears beyond a critical level of deformation (denoted by the strain *ε_f_*, where strain is defined as the length increase divided by the initial length), as shown in Figs. 2b. The deformation in the material decreases for strain beyond *ε_f_* by creating more fragments and decreasing their sizes. We measure the average fragment size as a function of strain as shown in Fig. 2c (detail in **Methods section**). It is shown that for strain beyond *ε_f_* the fragmentation size keeps decreasing, until it reaches a secondary critical deformation state (denoted by the strain *ε_c_*) when the pattern becomes stabilized and strain increases exclusively, leading to further opening the already-cracked edges of polygons. This results in an asymptotic fragment size. Using fracture mechanics theory, we derive how such fragmentation follows deformation by relating the deformation energy in the material to the surface energy used to create cracks (details see **Methods section**). We find that the fragment size during stretching can be expressed by: 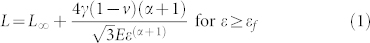
where *E* is the material's Young's modulus, *ν* is Poisson's ratio, 2*γ* is the fracture energy per unit area of the material, *ε* is the applied tensile strain caused by the mismatch between thin film and substrate, *L*_∞_ is the length of the residue scale, which is the asymptotic size and *α* is the stiffening factor that relates to the nonlinear material property of skin (detail in the Methods section). From this equation, we find that the evolution of the fragment size is governed by two factors: the applied strain as well as the ratio between the fracture energy and the material stiffness. The comparison of the importance of those two factors is adjusted by the nonlinearity given by *α*. We summarize some plausible values for 2*γ*/*E* for different skins (human hand, chicken and crocodile) measured in experiments as shown in Table 1. Those values are used to fit the simulation result as shown in Fig. 2c. We find that only small 2*γ*/*E* value, as is observed in crocodile skin, gives a good interpretation for the simulation result. From this analysis we also obtain the empirical value of *α* as 2.4, which corresponds to a stiffening hyper-elastic material, as common in nature. Moreover, we find the best fit with unphysical negative *L*_∞_ values for human hand and chicken, while physical positive *L*_∞_ value for a crocodile. This result suggests that it is the mechanical force and releasing of deformation energy that dominates the fragmentation of crocodile skin and makes the pattern eventually reach the stable characteristic length of *L*_∞_ (Fig. 1a). The negative *L*_∞_ value, in contrast, suggests the surface geometry of those skins is not caused by fragmentation, as they can form corrugated pattern with more complex geometry but not asymptotic size (until the cellular size) (as shown in Fig. 1c).

**Figure 3:**
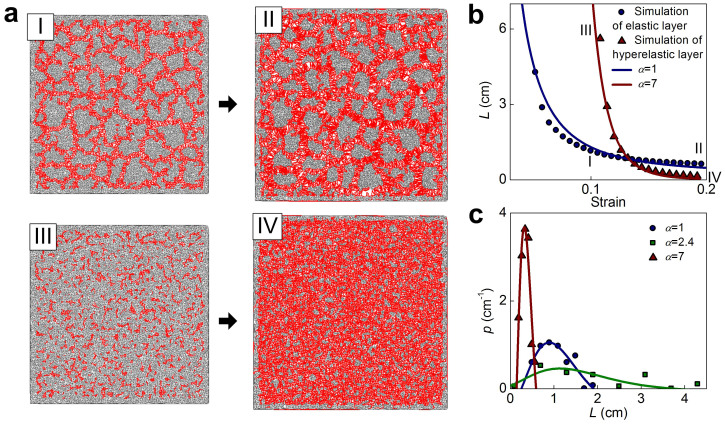
Fragmentation under biaxial tensile deformation for two materials with different nonlinearity. (a), The simulation snapshots of evolving cracking pattern in linear elastic (*α* = 1) material that is deformed at *ε* = 0.1 (I) and *ε* = 0.2 (II), and in hyper-elastic material (*α* = 7) with strong stiffening behavior that is deformed at *ε* = 0.1 (III) and *ε* = 0.2 (IV). All the loading conditions are same as what is used in Fig. 2 but the fragmentation patterns are significant different. (b), The fragment size as function of the increasing strain for the elastic two materials (*α* = 1 and 7). Data points I ~ IV correspond to the snapshot in a. It is shown that the fragmentation starts to appear in the linear elastic material at a strain (*ε_f_* = 0.05) smaller than the hyper-elastic material (*α* = 7 and 2.4 here and Fig. 2c, respectively). The hyper-elastic material with strong stiffening behavior (*α* = 7), once generates fragmentation at a larger strain, quickly forms a pattern with many small fragments (*L* = 0.2 cm). (c), The probability distribution (*p*) of fragment size measured from simulation snapshots of the three materials (*α* = 1, 2.4 and 7) at the end of simulation of *ε* = 0.2. Both *α* = 1 and 7 (1.0 ± 0.3 and 0.25 ± 0.1 cm, respectively) lead to more uniform and symmetric distribution of the fragment length than *α* = 2.4 (1.8 ± 1.1 cm) which contents some fragments much larger than the rest.

We change the mechanical characteristic of the network structure (changing from *α* = 2.4 to *α* = 1 and 7) by using different power exponent of the constituent springs and compare the characteristic nature of the fragmentation in those different systems, as shown in Fig. 3. It is seen that the fragmentation starts to appear in the linear elastic material (*α* = 1) at a smaller strain (*ε*_f_ = 0.05) than the hyper-elastic material and the hyper-elastic material with strong strain-stiffening characteristic (*α* = 7) generate the fragmentation at a much larger strain (Fig. 3b). Moreover, for *α* = 7, it is observed that the fragmentation starts with many disconnected small cracks as shown in Fig. 3a, suggesting that this strain-stiffening material is able to diffuse the deformation energy in the entire deformed region instead of concentrating at several crack tips^14^. Further increasing the deformation for this material quickly lead to the fragmentation pattern with many small fragments (*L* = 0.2 cm as shown in Fig. 3b). We measure each fragment length of the three materials (*α* = 1, 2.4 and 7) at the end of simulation of *ε* = 0.2 and obtain the probability distribution as shown in Fig. 3c. It is found that both *α* = 1 and 7 lead to smaller fragments with more uniform (*L*_max_/*L* = 1.9 and 1.8, for *α* = 1 and 7, respectively, where *L*_max_ is the maximum length and *L* is the average length of all the fragments) and symmetric distribution of the fragment length than *α* = 2.4 which contains fragments much larger than the rest (*L*_max_/*L* = 2.5). The result obtained for *α* = 1 quantitatively agrees with the experimental observation for oxide coating, which is taken as a linear-elastic material, as *L*_max_/*L* = 1.65 ~ 1.8 is obtained for this material under different applied strains^12^. These results agree with what we observe for crocodile skin and other thin films (Fig. 1) as the fragments in crocodile skin (Fig. 1a) are larger and have more irregular length than in linear-elastic material (ceramics and surface coating in Figs. 1f and b, respectively) or hyper-elastic material with strong strain-stiffening characteristic (human skin in Fig. 1c), suggesting again that the fragmentation pattern of crocodile skin is strictly governed by its mechanical properties.

**Figure 4:**
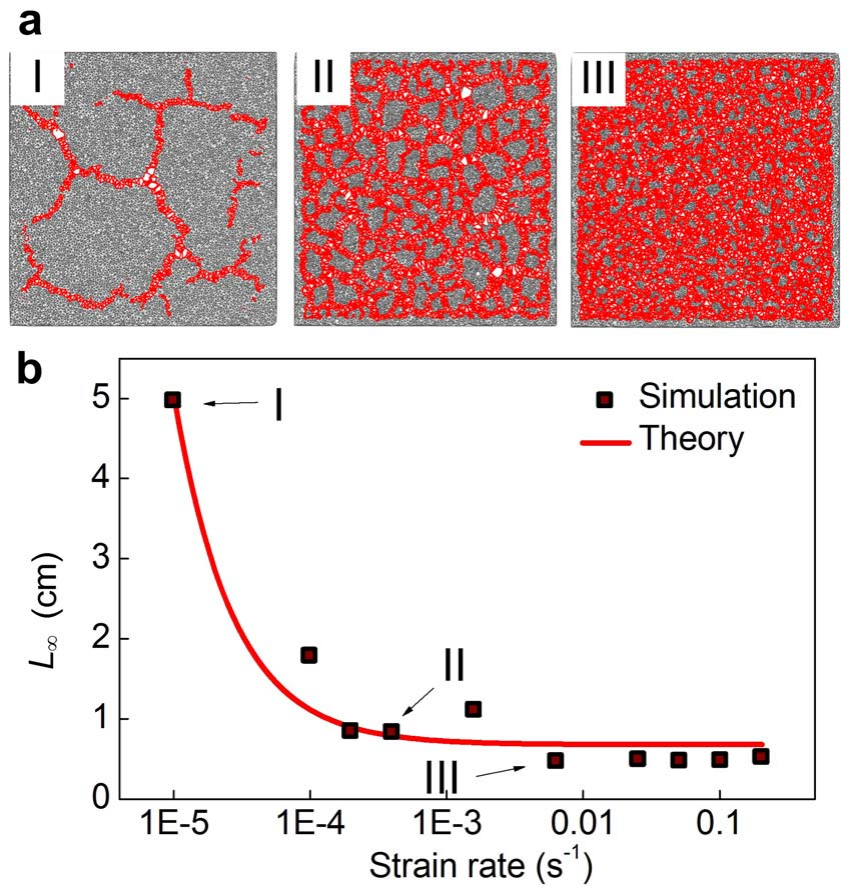
Illustration of the rate-dependent geometry under biaxial tensile deformation. (a), Snapshots of fragmentation patterns under different strain rates (I) slow, (II) medium, (III) fast. (b), Asymptotic size of polygons (*L*_∞_), when the material is fully fractured, as a function of the strain rate (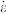), clearly showing a strain rate effect on the resulting pattern of the fragmentation geometry. The continuum curve is fitted according to 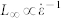. The result shows that the asymptotic size of fragmentation depends on the strain rate, which implies the fragmentation pattern is tunable according to the loading conditions.

**Figure 5:**
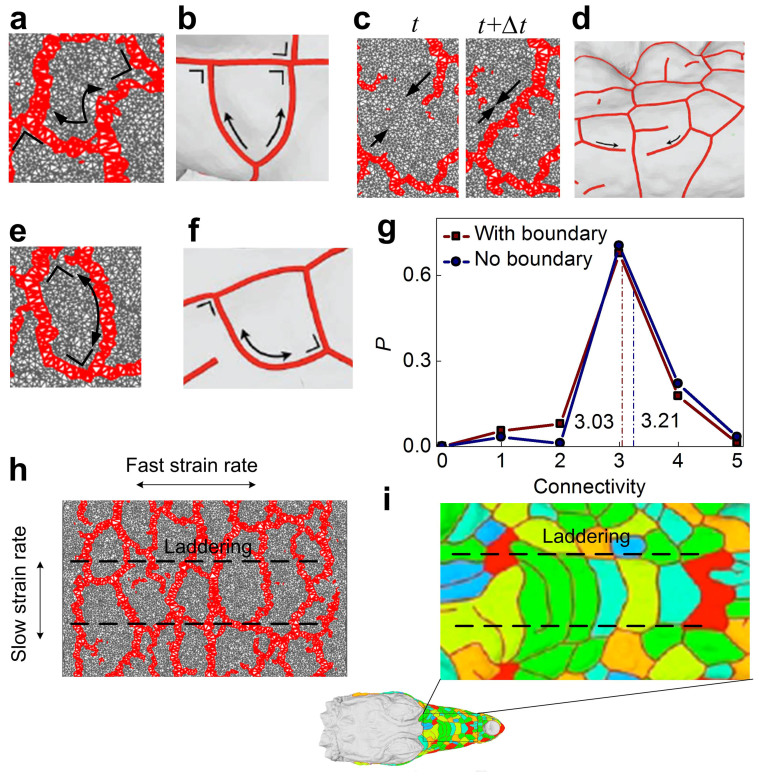
Comparison between the simulated fragmentation in a thin film and *in situ* observation of a crocodile's head^9^. (a), Branching of the crack predicted in simulation. (b), Branching of the crack of *in situ* observation. (c), merging of two cracks based on simulation of two sequencing time steps. (d), merging of two cracks of *in situ* observation. (e), The extension of a crack meets other cracks perpendicularly in simulation. (f), The extension of a crack meets other cracks perpendicularly of *in* *situ* observation. (g), Probability distribution (*P*) of vertices as a function of connectivity, which denotes the number of cracks connecting at each vertex, for polygons of the cracking pattern in simulation as the result for all polygons (red with square) and the result without boundary polygons (blue with circle). Average values are indicated by dash lines. (h), The laddering pattern induced by heterogeneous strain rates in simulation. (i), The laddering pattern of *in situ* observation on the top of crocodile's head. All *in situ* observation images (b), (d), (f), (i) are reproduced from^9^. The geometry of crocodile's head scales shows similar characteristic as what is seen in the simulation, suggesting that our model captures the mechanism of fragmentation of crocodile skin.

We now change the strain rate and investigate how the deformation speed alters the fragmentation pattern. The stabilized patterns under multiple strain rates are as shown in Fig. 4a, where it is demonstrated that a faster strain rate induces more fragments with smaller size. The asymptotic size of polygons *L*_∞_, as systematically shown in Fig. 4b, more clearly illustrates that the fragmentation is strongly rate-dependent at small deformation rate while becomes insensitive of large deformation rate. By considering—as posed by classical viscoelasticity—the scaling *E*(*t*) ~ *E*(*t* = 0)/(*t*/*τ* + 1) (*τ* is a characteristic time)^15^, since 
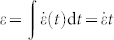
 where *t* is the time for applying deformation of constant strain rate, assuming the asymptotic size is proportional to the fragment size at largest strain we obtain that 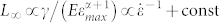 (here *ε_max_* > *ε_c_* is the applied strain for obtaining asymptotic fragment size). This relationship is used to fit the simulation results as shown in Fig. 3b. Indeed, this mechanism, combined with the anisotropic growth of the head skull, explains the anisotropic and irregular fragmentation pattern seen in crocodiles (Figs. 5h and i). This result also suggests that the fragmentation pattern varies for paints because of the different deforming history caused by environmental change, generating the unique fingerprint on each of them. It is also noted that for small loading rate, the fragment size approximates exponential scaling, but the size departs from the exponential at larger loading rate because the resolution of the network structure for generating the fragments is limited by the initial length of the springs in the model, which has the physical meaning of the size of the constituent particles such as cellular size of the skin. This factor can be involved by combining experimental measurements of cellular size to improve the biological insight of the model in future.

**Figure 6:**
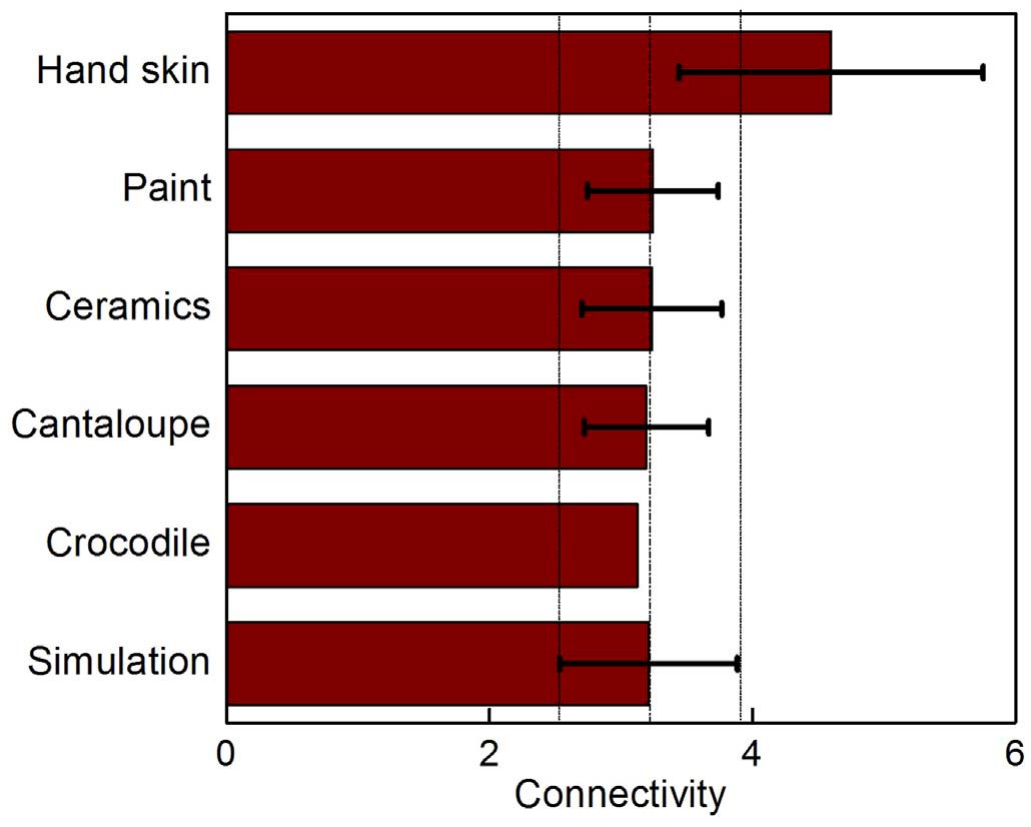
Comparison of the connectivity of the fragmentation patterns of different systems. Average values are given by main bars and the standard deviations are given by error bars. The simulation result is extracted from Fig. 4g without boundary polygons and its mean value and standard deviation are highlighted by dash lines. The agreement of the connectivity observed in simulation and other systems including crocodile, cantaloupe, ceramics and paint. The connectivity of hand skin shows significant different value. This result suggests that the mechanism revealed by our model explains the fragmentation of thin films on several different systems, while it does not account for generating the pattern on hand skin (perhaps because active cellular processes are involved).

We systematically compare the simulation result against *in situ* observations of the fragmentation of crocodile head, as illustrated in Fig. 5. It is clearly seen that the emerging fragmentation pattern very well reproduce what is observed on the crocodile head. The connectivity of the polygons has an average value of 3.03 to 3.21 (with and without boundary), and this connectivity range yields a mean number of sides of polygons between 5.88 and 5.31. This is in excellent agreement with the experimental observation of 5.532 ref. [9], and indicates that polygons are nearly hexagonal and those closer to the boundary have more sides. Moreover, the model shows that introducing heterogeneous deformation—considering that the strain rate (or bone growth) in one direction is faster than that in the other, orthogonal direction–captures the characteristic laddering shape on the top of crocodile's head as illustrated in Figs. 5h and i. This finding demonstrates that the laddering scale pattern observed on crocodile head is caused by the fact that the head's longitudinal growth is faster than in other directions^9^,^16^. To demonstrate the significance of the fragmentation of crocodile, we summarize the connectivity of the fragmentation pattern of different systems as shown in Fig. 6. It is shown that the fragmentation of crocodile head has similar geometry as is observed in cantaloupe, ceramics and paint and their patterns are all captured by our mechanics model. The significant difference for the skin crumpling of hand skin indicates that the corrugated pattern with complex geometry is governed by involving other mechanisms rather than tension-induced fragmentation.

## Discussion

In this study, we identify two critical strains (*ε_f_* for initiating of fragmentation and *ε_c_* for reaching the asymptotic fragment size), which are induced by skeletal growth, that govern the initial and final mechanics of skin fragmentation. Those two critical strains divide the deformation procedure into three regimes, which agrees with what is observed in the cracking of other thin film systems^17^, except that our results do not statistically depend on the initial random distribution of defects. The first critical strain explains why there is no fragments found before 45 days of embryonic crocodile, as is found by *in situ* observation^9^, likely because the accumulated strain remains below the threshold (*ε_f_*) during that period. The second critical strain explains why the pattern does not change (or re-develop anew) repeatedly over time, as no new polygons are created by the increasing strain above the other threshold (*ε_c_*). Our result also shows that the fragmentation depends on the ratio between the fracture energy and Young's modulus of the skin. The mechanical property of crocodile skin makes it unique to form the polygon pattern during development. Flexible skins of other animals, such as mammal and bird, are hard to reach a stable fragmentation pattern in the skin under normal state. However, there are extreme conditions^18^, for example dehydration, oldness and illness^19^,^20^,^21^,^22^, which can alter the mechanics of the skin and make the stable fragmentation possible. From a different angle of view, the skin fragmentation of crocodile can be attributed to mechanical adaption^23^. The hard skin of crocodile with fragmentation can greatly increase the flexibility (with reduced bending stiffness because cracks set fragments free-by reduced entropic elasticity^24^-to move out of plane) and reduce the difficulty for movements at the end of jaw. A similar strategy is also observed to be adopted by armoured fish as its discrete scales for body protection and mobility^20^,^25^.

The fragmentation on crocodile heads is unique for animal skins but shows a similar geometry as many inorganic thin films. Here, we investigated the mechanism hidden behind these phenomena by introducing a mechanical model and probing the entire fragmentation process. Our results demonstrate that the ratio between the fracture energy and Young's modulus together with deformation rate governs the characteristic size of the fragmentation pattern. By using this knowledge learnt from crocodile skin, we may design and produce surface coating with improved stability by using synthetic materials. For instance, we may use heterogeneous distribution of stiff and soft materials, which mimic the crocodile scales and their joints, for coating under the help of digital manufacturing technologies. By using the strategies one can make the coating stable from further fragmentation and hence have longer lifetime and provide more efficient protection.

## Methods

### Details of the elastic network model

The model we use to simulate the fragmentation behavior of keratinized epidermal under biaxial tension is a simple elastic network model. The model is composed of a collection of beads and inter-bead connections ref. [14,26]. The initial coordinates of all the beads are randomly distributed. The topology of the inter-bead connection is designed by Delaunay triangulation algorithm^27^. This method enables generating triangle meshwork from randomly distributed point and maximizes the minimum angle of all the angles of the triangles, avoiding irregular triangles. For each inter-bead connection in the triangulation, we use a breakable nonlinear springs to represent the interaction. The tensile force for each spring is given by *f* = *δk*_0_(Δ*r*/*r*_0_)*^α^* where *r*_0_ is the initial length of the spring, Δ*r* is the deformation of the spring, *k*_0_ is the stiffness of a unit length spring, *α* is the stiffening factor that relates to the nonlinear property of the spring, *δ* is a cut off function with its value given by 
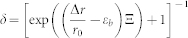
 with 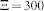 as the smooth factor and *ε_b_* = 0.05 as the bond breaking strain. It is noted that by using this force function, the stiffness of each spring stochastically varies with its initial length, ensuring the uniform mechanics of the material. Without loss of generality, all parameters in the model, including bead mass, spring stiffness and film length are set unitless (without any fitting). We apply biaxial tensile strain to deform the entire film with an overall constant strain rates. Yet the deformation is applied in a discrete from: for every a hundred steps, we add the constant strains to the film at the first step and equilibrate the film during the rest steps with the single layer of beads at the four edges fixed. This loading method mimics the tension field caused by skeletal growth and applied on the epidermal on the crocodile head as illustrated in Fig. 1g (considering that the bone is 1–2 orders of magnitude stiffer than the epidermal and it can be modeled as a rigid substrate to generate homogenous deformation to the epidermal in every single direction^28^). This model enables us to systematically investigate how growth history and growth rate are coupled^29^ to determine the evolution of fragmentation pattern.

### Monitoring fragmentation and computing its size in the simulation

During the post-analysis of the simulation we monitor the fragmentation pattern as assemblies of polygons by identifying all rupture springs (Δ*r*/*r*_0_ ≥ 0.05) and highlight them by taking snapshots. To compute the size of those polygons, we simply assume the cracks are in form of hexagonal lattices and the total lattice area is given by 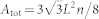 where *n* is the number of scales and *L*/2 is length of the hexagonal edge (*L* is the scale size), while the area of the polygon edge with width 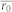 (the average spring length at equilibrium) is 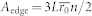. Noting that the ratio between the area of the polygon edge and total surface area equals the ratio between the number of beads involved in broken bonds (*N_crack_* as a function of strain) and the total bead number (*N*). These relations enable us to obtain the fragment size according to 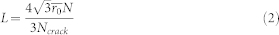
We have measured the distribution of fragment size for cases *α* = 1, 2.4 and 7 and summarized the result in Fig. 3c. It is shown that the average value and standard deviation of the fragment is 1.0 ± 0.3, 1.8 ± 1.1 and 0.25 ± 0.1 cm for *α* = 1, 2.4 and 7 respectively, which agrees with the measurement given by using Eq. 2 as 0.8, 1.2 and 0.2 cm for *α* = 1, 2.4 and 7, respectively as shown in Figs. 2c and 3b.

### Fracture mechanics analysis for the fragmentation size

Here we derive how fragmentation size follows deformation. The energy balance during crack propagation imposes that the variation of the total potential energy dΠ, equal to the variation of the elastic strain energy dΦ minus the variation of the external work dΨ, must be equal to the opposite of the work spent in creating the new surface of fragments d*S*, *i.e* dΠ = dΦ − dΨ = −2*γ*d*S*, where 2*γ* is the fracture energy per unit area. Assuming linearity for the constitutive law of the film implies the validity of the Clapeyron's theorem^30^, *i.e.* dΦ = dΨ/2. Accordingly, dΠ = −dΦ and the condition for fragmentation becomes dΦ = 2*γ*d*S*. Note that this result is in general valid also for nonlinear systems under imposed displacements; for this case, in fact, the external work is identically zero and thus we do not need to apply the Clapeyron's theorem to obtain it. By integration, we have ΔΦ = 2*γ*Δ*S*, where Δ*S* is the new crack surface area created as Δ*S* = 3/2*nLb* (for hexagonal fragmentation geometry) where *n* is the number of scales, *b* is their thickness and *L* is their size. We now generalize the stress-strain relation as *σ* = *Eε^α^* by considering the nonlinearity of materials and estimate the deformation energy as ΔΦ = 2*AbEε*^(*α*+1)^/(1−*ν*)/(*α* + 1), where *ε* is the accumulated mismatch biaxial strain between skin and skeleton, *A* is the film surface area, *b* is its thickness, *E* is the secant modulus that equals to Young's modulus of linear material or general materials at small deformation, *ν* is Poisson's ratio and *α* is the stiffening factor that relates to the nonlinear material property of skin (α < 1 for elastic-plastic, α = 1 for linear, α > 1 for hyper-elastic material). Accordingly, the fragmentation is expected when the following system holds: 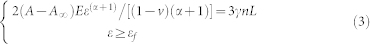
where *ε_f_* is the skin fracture strain as shown in computational modelling and *A*_∞_ is the residue area that still subjects to deformation after fragmentation that we assume to be proportional to the fragment perimeter where the residual deformations concentrate^31^. Accordingly, since for the hexagonal geometry 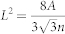, we can define 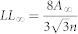 and the fragment size during stretching is given by Eq. (1).

## Author Contributions

Z.Q., N.M.P. and M.J.B. designed the research. Z.Q. implemented the computational model and analysis tools, carried out the simulations and collected the data. N.M.P. and Z.Q. performed the theoretical analysis and collected the data. Z.Q., N.M.P. and M.J.B. analyzed the results and wrote the paper.

## Figures and Tables

**Table 1:** Fracture energy (2*γ*) and Young's modulus (*E*) of skin of different animals

Skin type	Fracture energy (2*γ*) (kJ/m^2^)	Young's modulus (*E*) (MPa)	Ratio (2*γ/E*) (m)
**Human hand skin [19,32]**	1.8	0.6	0.003
**Chicken skin [33]**	2.8	8	0.0004
**Crocodile skin (hard keratin) [34]**	10	5600	0.000002
